# Relevance of PTEN loss in brain metastasis formation in breast cancer patients

**DOI:** 10.1186/bcr3150

**Published:** 2012-03-19

**Authors:** Harriet Wikman, Katrin Lamszus, Niclas Detels, Liubov Uslar, Michaela Wrage, Christian Benner, Ina Hohensee, Bauke Ylstra, Kathrin Eylmann, Marc Zapatka, Guido Sauter, Dirk Kemming, Markus Glatzel, Volkmar Müller, Manfred Westphal, Klaus Pantel

**Affiliations:** 1 Institute of Tumor Biology, University Medical Center Hamburg-Eppendorf, Martinistreet 52, 20246 Hamburg, Germany; 2 Department of Neurosurgery, University Medical Center Hamburg-Eppendorf, Martinistreet 52, 20246 Hamburg, Germany; 3 Departments of Pathology and Otolaryngology, Head and Neck Surgery, VU University Medical Center, De Boelelaan 1118, 1081 HZ Amsterdam, The Netherlands; 4 Division of Theoretical Bioinformatics, German Cancer Research Center, Im Neuenheimer Feld 280, 69120 Heidelberg, Germany; 5 Institute of Pathology, University Medical Center Hamburg-Eppendorf, Martinistreet 52, 20246 Hamburg, Germany; 6 European Laboratory Association, Roggenkampstrasse 12, 49477 Ibbenbueren, Germany; 7 Department of Neuropathology, Medical Center Hamburg-Eppendorf, Martinistreet 52, 20246 Hamburg, Germany; 8 Department of Gynecology, University Medical Center Hamburg-Eppendorf, Martinistreet 52, 20246 Hamburg, Germany

## Abstract

**Introduction:**

With the improvement of therapeutic options for the treatment of breast cancer, the development of brain metastases has become a major limitation to life expectancy in many patients. Therefore, our aim was to identify molecular markers associated with the development of brain metastases in breast cancer.

**Methods:**

Patterns of chromosomal aberrations in primary breast tumors and brain metastases were compared with array-comparative genetic hybridization (CGH). The most significant region was further characterized in more detail by microsatellite and gene-expression analysis, and finally, the possible target gene was screened for mutations.

**Results:**

The array CGH results showed that brain metastases, in general, display similar chromosomal aberrations as do primary tumors, but with a notably higher frequency. Statistically significant differences were found at nine different chromosomal loci, with a gain and amplification of *EGFR *(7p11.2) and a loss of 10q22.3-qter being among the most significant aberrations in brain metastases (*P *< 0.01; false discovery rate (fdr) < 0.04). Allelic imbalance (AI) patterns at 10q were further verified in 77 unmatched primary tumors and 21 brain metastases. AI at *PTEN *loci was found significantly more often in brain metastases (52%) and primary tumors with a brain relapse (59%) compared with primary tumors from patients without relapse (18%; *P *= 0.003) or relapse other than brain tumors (12%; *P *= 0.006). Loss of *PTEN *was especially frequent in HER2-negative brain metastases (64%). Furthermore, *PTEN *mRNA expression was significantly downregulated in brain metastases compared with primary tumors, and *PTEN *mutations were frequently found in brain metastases.

**Conclusions:**

These results demonstrate that brain metastases often show very complex genomic-aberration patterns, suggesting a potential role of PTEN and EGFR in brain metastasis formation.

## Introduction

Breast cancer is the most common malignancy in women, with the mortality rate being especially high in patients in whom brain metastases develop. Approximately 15% to 20% of breast cancers metastasize to the brain, although incidence rates are increasing [1]. The incidence of metastases is thought to be increasing because of the improved treatment of metastases at other distant sites and advances in imaging techniques, leading to improved detection of central nervous system (CNS) metastases [2].

Metastasis formation is a highly selective, multistep process, involving complex interactions between tumor and host cells. To metastasize, tumor cells must disengage from the primary tumor, invade the stroma, and penetrate into vessels, where they disseminate, extravasate, and start to grow at distant organ sites. As a distant metastatic site, the brain forms a special challenge for tumor cells because of the blood-brain barrier [3]. In addition, all other steps have to be successfully completed for the tumor cell to survive and expand. The molecular basis for all of these steps is still unclear, and several models have been suggested [4,5].

Comparative gene-expression analyses on primary breast tumors and lymph node metastases have indicated that, in general, metastases have very similar expression signatures compared with their parent tumors [6,7]. However, detailed analyses have also revealed that a number of genes are consistently differentially expressed between primary tumors and metastases [8-10] and that metastases often show a greater variety of aberrations than the primary tumor [11,12]. At the chromosomal level, even greater differences have been described between primary breast tumors and their derived metastases. Most of the relevant studies compared the chromosomal aberrations in matched primary breast tumors and lymph node metastases [13-16], and only a very few studies on distant metastases exist [17-19]. In general, all of these studies showed that metastases harbor more and also new aberrations that could not be found in the corresponding primary tumors (reviewed in [20]). These results imply that the clonal evolution of a tumor is more complex than would be predicted by linear models, highlighting the importance of investigating distant metastases as the end point of the metastatic cascade.

In this study, the patterns of chromosomal aberrations of primary tumors and brain metastases from breast cancer patients were compared with array-comparative genomic hybridization (CGH) and microsatellite analysis. The goal was to identify genetic alterations in the primary breast tumors associated with metastatic spread to the brain to be able to define subgroups of high-risk breast cancer patients. Our results indicate that loss of 10q and especially phosphatase and tensin homologue (*PTEN*) could be predictive factors for the development of brain metastases. Interestingly, whereas loss of PTEN is generally very rarely seen in most epithelial tumors, it is one of the most frequent aberrations found in primary glioblastomas [21] and other CNS malignancies [22,23], indicating that loss of PTEN might be an important factor for breast tumor cell survival in the CNS environment.

## Materials and methods

### Patient collection

All samples were collected from female patients who underwent surgical resection at the University Medical Center, Hamburg-Eppendorf, Germany. For array CGH profiling, unmatched fresh-frozen tumor samples were collected from 30 primary breast cancer patients, with 10 breast cancer samples that had metastasized to the brain. All primary tumors were of an early stage, and none relapsed to the brain at a later stage (mean follow-up of 58.8 months). The mean age of the patients at brain metastases surgery was 57 years, with an average of 11 years (range, 4 to 30 years) between primary tumor diagnosis and brain relapse (Additional file 1).

For allelic-imbalance (AI) analysis, 77 primary breast tumor (55 fresh-frozen and 22 paraffin-embedded samples) and 21 brain metastases (all fresh-frozen) samples were analyzed. To avoid misleading results by analyzing unmatched samples, the primary tumor samples were matched for the main clinicopathologic characteristics in the AI analysis. Ten of the primary tumors later showed a relapse to the brain, and 17, to other sites. Eighteen of the primary tumor cases and eight metastases overlapped with the array samples, and five pairs of primary and corresponding metastases were available (Additional file 1). Four pairs of matched primary tumor and brain metastases were also available.

This study was approved by the Ethics Committee of the Chamber of Physicians, Hamburg, Germany, and sample donors gave written informed consent.

### HER2 status

The HER2 status was first assessed in both the primary tumors and the brain metastases with immunohistochemical tests (Dako HercepTest; Dako, Glostrup, Denmark) according to the manufacturer's instructions. All cases with an immunoscore of 3+ were considered to be HER2 positive, whereas all cases with a score of 2+ were reevaluated with fluorescence *in situ *hybridization (FISH) (Path-Vysion Kit Vysis; Abbott, Abbott Park, IL, USA). Tumor tissue in which the HER2-FISH signal to centromere 17 ratio was > 2 was also considered HER2 positive.

### Array CGH

Genomic DNA was isolated (QIAmpDNA MicroKit; Qiagen, Hilden, Germany) from fresh cryosections for the array CGH analysis and part of the microsatellite analysis. If necessary, manual microdissection was performed to obtain a tumor-cell content of at least 70% [24]. Total volumes of 300 ng of tumor DNA and 300 ng of reference DNA (pooled leukocyte DNA of 10 healthy men) were labeled with Cy3- and Cy5-labeled dCTP, respectively, and co-hybridized on 30 K oligonucleotide CGH microarrays. The array contains 60 mer oligonucleotides representing 28,830 unique genes designed by Compugen (Human Release 2.0 oligonucleotide library; San Jose, CA, USA) [25]. The raw signal intensities were obtained by using Agilent's Feature Extraction software program after bad-quality spots were removed by using BlueFuse Version 3.1 (BlueGenome, Cambridge, UK). We used a sex-mismatch in the hybridization (that is, DNA of the opposite gender was used as a reference). Subsequently, the X and Y chromosomes were excluded for downstream data analysis. The log2 ratios were centered to a median of zero, and the resulting log2 ratio values for each probe were segmented by using GLAD in R [26]. All probes within the genomic bounds of a given GLAD-derived segment were given the mean copy-number value of the probes within that segment. Copy-number values > 0.05 log2 ratio units represented a gain and values < -0.05 log2 ratio units represented a loss. The cut-off points were based on the variance found in the X and Y reference chromosomes. After segmentation, the log2 ratios were centered to a median of zero by using only normal segments, and the segmentation was repeated. All CGH data are available at ArrayExpress [27].

### Microsatellite analysis

Microsatellite analysis was carried out to verify the loss of 10q and to reveal the extent of the aberration in 77 primary and 21 brain metastases. Tumor DNA was isolated in 22 cases from paraffin-embedded samples, also using macrodissection to achieve a minimum of 70% tumor cells in the samples. The DNA was extracted by using the InnuPREP DNA Microkit (AnalytikJena, Jena, Germany). For reference DNA, DNA samples isolated from peripheral blood mononuclear cells or nonmalignant normal breast tissue were used. Allelic imbalance in the chromosomal region 10q22.3-qter (chr10:80,522,854-134,299,490; 53.8 MBp) was first assessed by using eight microsatellite markers with an approximate spacing of 10 MBp. Three additional microsatellite markers were analyzed in samples that contained enough DNA and showed a partial AI (Additional file 2). The FAM or HEX end-labeled polymerase chain reaction (PCR) products were analyzed with a Genetic Analyzer 3130 (Applied Biosystems, Darmstadt, Germany). GeneScan software (Applied Biosystems) was used to study the lengths of the allele fragments and fluorescence intensity. The allelic imbalance (AI) was determined for heterozygous markers by calculating the ratio of the peak heights of the tumor and normal alleles. Ratios of 2.0 or higher were scored as AI.

### *PTEN *mutation analysis

For the 10 brain metastases samples, the entire *PTEN *coding region was screened for mutations by sequencing PTEN cDNA. For an additional 10 samples, exons 3 and 5 were sequenced for mutations (Additional file 2). For the sequencing of cDNA total RNA was extracted from fresh-frozen tissue by using the Qiagen Minikit (Qiagen, Hilden, Germany), and then the total RNA was reverse transcribed by using a First Strand cDNA Synthesis Kit (Fermentas, St. Leon-Rot, Germany). First, the complete coding region was amplified, followed by sequencing PCR for both DNA strands. When multiple PCR products were detected, the respective bands were gel-purified by using the GelExtract Mini Kit (5Prime, Hamburg, Germany), and 40 ng of the purified product was used for the sequencing PCR by using the BigDye Terminator v1.1 Cycle Sequencing Kit (Applied Biosystems, Freiburg, Germany). For genomic DNA, PCRs were performed by using exon-spanning primer pairs for exons 3 and 5, as described in Danielsen *et al*. [28]. The PCR products were purified by using the PCRExtract Mini Kit (5Prime), and subsequent sequencing PCR was performed by using 30 ng of DNA. The sequences were determined in a Genetic Analyzer 3130 (Applied Biosystems).

### Gene-expression analysis of the 10q gene in primary tumors and brain metastases

Array data from the 32 untreated primary breast tumors without relapse included in GEO DataSet [GSE21974] (including 10 basal-like and 22 non-basal-like tumors) and nine brain metastases samples (the same samples as used for CGH analysis, except for one sample from which no RNA could be extracted) were compared for differentially expressed genes. The datasets, which both were analyzed on the Agilent Whole Human Genome Microarray 4 × 44K, were combined, quantile normalized, and checked for systematic differences between the two array groups. Subsequently, differentially expressed genes were selected by using the significance analysis of microarrays (SAM) algorithm with a false-discovery rate (fdr) of 5%. To narrow the results, in a second step, only transcripts with an expression level at the 25th percentile or greater of the overall expression level, located on chromosome 10 with a fold change (FC) > 2, were taken into account.

In addition, a data set from Zhang *et al*. [11] GEO DataSet [GSE14020] was analyzed to see whether a difference in the PTEN expression exists among different primary tumor patients with different relapse patterns. The data set consist of primary breast tumors with 22 cases of brain relapse, 20 cases with lung relapse, and 18 cases with bone relapse. The CEL files were processed by using GCRMA. Differentially expressed genes (brain versus bone relapse and brain versus lung relapse) were identified by repeated permutation testing with the SAM algorithm by using a 5% fdr.

### Statistical analysis

Statistical analyses were performed within the R statistical environment. For array CGH data, differences between primary tumors and brain metastases with respect to copy-number changes were determined per probe region by using CGHMultiarray, which is based on the Wilcoxon rank-sum test and is implemented in the CGHtest R-package [29,30]. Raw *P *values were corrected for multiple testing by using Benjamini and Hochberg's False Discovery Rate method [31].

The relation between microsatellite markers and clinical factors was examined by means of the χ^2 ^test and of independence. Differences between primary tumors and brain metastases in relation to allelic imbalance were calculated with the Fisher Exact test.

## Results

### Comparative analysis of primary and metastatic breast tumors with array CGH

**Figure 1:**
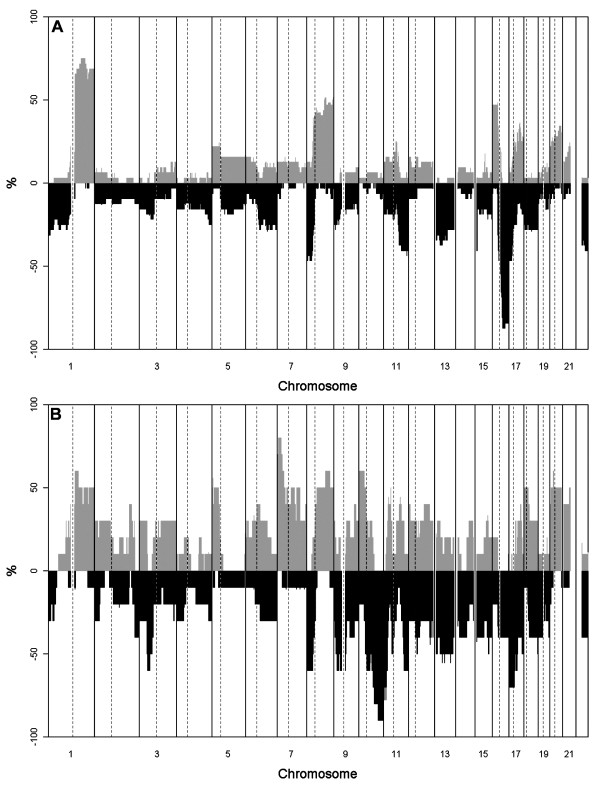
**Frequency plot of the CGH results**. In primary **(a) **breast tumors and brain metastases **(b)**, The positive Y axis shows the percentage of patient samples with gains, and the negative Y axis shows the percentage of patient samples with losses. Chromosomes are ordered from 1 to 22 on the X axis. Chromosomal borders are marked with solid vertical lines, and centromere positions, with dotted lines.

To identify chromosomal aberrations that function as molecular markers for brain metastasis, array CGH screening was performed on both primary breast tumors and brain metastases. Copy-number changes were found in all samples (*n *= 40) and on each chromosome arm. The most frequently observed gains (> 30% of cases) in the primary tumors were found at 1q, 8q, 16p, 17, and 20, and the most common losses were at 1p, 8p, 11q, 13, 16q, 17p, and 22 (Figure 1a). In the brain metastases, a high degree of aberration was found on almost every chromosome, with the most common gains (> 30% of cases) at 1q, 5p, 7, 8q, 10p, 11p, 12p, 15q, 18, 20, and 22, and losses at 3p, 8p, 9p, 10q, 11, 12p, 13, 15, 17, and 18q (Figure 1b).

The results showed a very similar pattern of genetic aberrations in both primary and metastatic breast tumors (that is, the metastases carried the majority of genetic alterations present in the corresponding primary tumors but with a significantly higher frequency). Whereas 20 loci were gained or amplified and 17 lost in brain metastases (with > 30% difference), only gains of 1p and 16p and a loss of 16q were most frequently found in the primary tumors.

After correction for multiple testing (fdr < 0.04), nine regions were found to differ significantly (*P *< 0.05) between the primary tumors and metastases (Table 1). The most striking difference between the primary tumors and metastases was found at chromosome 10q, showing a loss of 10qter in 60% to 90% of the metastases, whereas only none to 13% of the primary tumors harbored a loss (*P *< 0.002). The terminal arm of chromosome 10p was often gained in the metastases (40%) compared with primary tumors, where no such gain was found (none) (*P *= 0.005). Gains of 7p22.2-p15.3 and 7p11.2 (EGFR) were found in 70% to 80% of the metastases but in only 10% to 13% of the primary tumors (*P *= 0.001 to 0.003, respectively). Interestingly, deletion at chromosome 17 in the primary tumors involved only the p-arm, whereas in brain metastases, most of the q-arm also was involved, except for the HER2 locus.

**Table 1:** Statistically significantly different regions between primary breast tumors and brain metastases

**Chrom. region**^**a**^		bp start	bp end	Size (Mbp)	% del PT	% gain PT	% del MET	% gain MET	*P *value	Fdr
**1**	p22.1-21.2	94472000	101828000	7.4	25.8	3.2	0.0	20.0	0.005	0.04
										
**7**	p22.1-15.2	5383000	27787000	22.4	3.2-12.9	12.9	0.0	70.0-80.0	0.001-0.002	0.01-0.02
	p11.2	54565000	55475001	0.5	0.0	9.7-12.9	0.0	70.0	0.001-0.003	0.01-0.02
										
**10**	pter-p12.1	173000	28895000	28.7	0.0	0.0	20.0	40.0	0.005	0.04
	q11.22-q21.1	49685000	59935000	10.3	3.2	6.5	60.0	20.0	0.005	0.04
	q22.1-qter	71226000	134848000	63.6	0-12.9	3.2-6.5	60.0-90.0	0.0-10.0	0.001-0.002	0.02
										
**11**	pter-p15.4	188000	9693000	9.5	12.9-19.4	9.7-12.9	70.0	0.0	0.001-0.005	0.01-0.04
										
**16**	q24.2	87416000	87465000	0.0	87.1	0.0	30.0	0.0	0.002	0.02
										
**17**	q11.2	26591000	29500000	2.9	16.1-22.6	25.8-29.0	70.0	0.0	0.001-0.005	0.01-0.04

fdr, false discovery rate; MET, brain metastasis; PT, primary tumor. ^a^The complete region that was statistically significantly different between the two groups (primary versus metastasis). Therefore, some break points in some patients might occur within the region, causing a variation in the frequencies of deletions/amplifications within a region and *P *values and fdr.

Twenty loci in the primary tumors showed a high-level amplification, whereas in the brain metastases, 10 loci harbored a high-level amplification. In general, no significant difference could be found in the number and distribution of high-level amplifications between the primary and metastatic tumors. The most common high-level amplification was the *HER2 *amplification at 17q21, which was found in 6% of primary and 20% of metastatic tumors (Additional file 3).

### Microsatellite analysis at 10q

**Figure 2:**
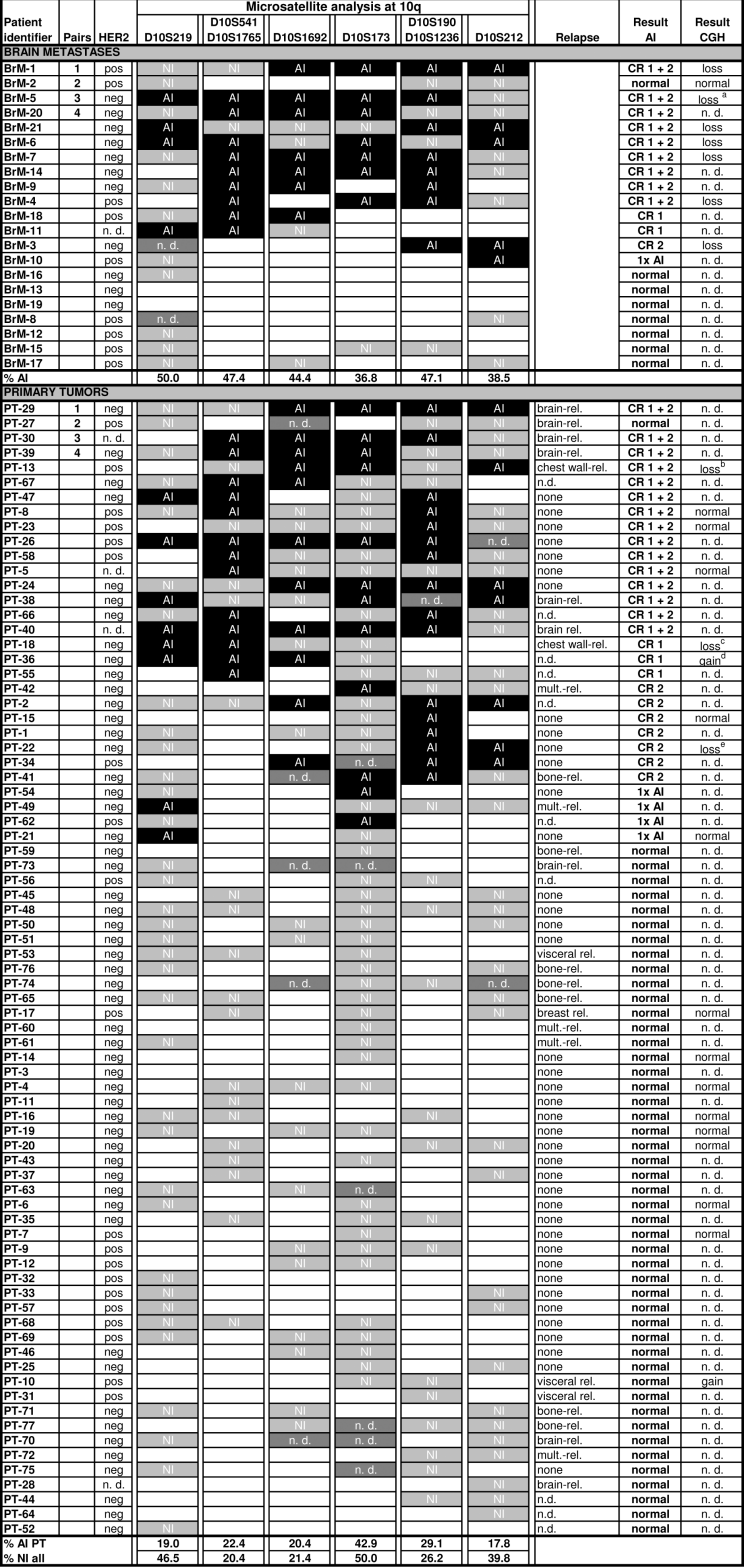
**Microsatellite analyses for AI on 10q in primary breast cancers and metastases**. Base-pair position and the markers used are indicated on the top line. The result for each marker is shown as follows: AI, black; noninformative, light gray; unavailable measurement, dark gray; and informative without changes, white box. 1, 2, 3, 4, matching primary tumors and brain metastases; a: loss from bp:100000000; b: loss from bp: 95000000; c: loss from bp:114000000; d: gain until bp:100000000; e: loss from 80-90000000; n.d., not determined; NI, noninformative marker; CR 1, core region 1; allelic imbalance around makers D10S173 and D10S190; CR 2, core region 2; allelic imbalance around markers D10S541 and D10S1765.

Microsatellite analyses for allelic imbalances (AIs) were carried out to verify the CGH results and to assess the extent of loss on 10q. Between eight and 11 microsatellite markers spanning a 54-MBp region on 10q were used to screen a total of 21 brain metastasis and 77 primary tumor samples, including four matched pairs of primary tumors and metastases (Figure 2). The primary tumor cohort was matched for the main clinicopathologic characteristics.

Significant differences in AI frequencies were found between the brain metastases and primary tumors without relapse (*P *= 0.05) (Table 2): 62% of brain metastases and 38% of the primary breast tumors were found to be carriers of AI in the 10q region. Interestingly, samples from primary tumors without a history of subsequent brain relapse or from patients with a relapse to organs other than the brain (28% and 18%, respectively) showed fewer AI than did primary tumors from patients with relapse to the brain (50%).

**Table 2:** Frequencies and *P *values for AI at chromosome 10q in primary breast tumors and metastases

	Brain metastases (*n *= 21)		**All primary tumors (*n *= 77)**^**a**^			Primary tumors without relapse (*n *= 39)			Primary tumors with brain relapse (*n *= 10)			Primary tumors with other relapse (*n *= 17)		
	
	*n*	%	*n*	%	***P *value**^**b**^	*n*	%	***P *value**^**b**^	*n*	%	***P *value**^**b**^	*n*	%	***P *value**^**b**^
**PTEN (CR1)**^**c**^	11	52.4	18	23.4	**0.006**	7	17.9	**0.003**	5	50.0	n.s.	2	11.8	**0.006**
**CR2**^**c**^	10	47.6	22	28.6	n.s.	11	28.2	n.s.	5	50.0	n.s.	3	17.6	n.s.
**all AI**	13	61.9	29	37.7	n.s.	13	33.3	0.055	5	50.0	n.s.	5	29.4	n.s.
**normal**	8	38.1	48	62.3	-	26	66.7	-	5	50.0	-	12	70.6	-

^a^Relapse pattern not recorded for 11 patients. ^b^*P *values calculated with the Fisher Exact test (brain metastases versus other groups). ^c^CR1 and CR2 are included in all AI. AI, allelic imbalance; CR2, core region 2; allelic imbalance around markers D10S190 and D10S1236; n.s., not significant; *PTEN *(CR1), core region 1 allelic imbalance around markers D10S1765 and D10S541.

The frequency of AI for individual markers varied between 18% and 43% in the primary tumors and 37% and 50% in the brain metastases. Interestingly, the AI did not cover the entire region but was concentrated around two core regions (Additional file 4). The first core region (CR1) was found around the *PTEN *locus (markers D10S541 and D10S1765), and the second region (CR2) was detected around markers D10S173 and D10S190. In particular, the AI in the CR1 (around the *PTEN*) locus was observed significantly more often in brain metastases than in primary tumors from patients without later relapse (*P *= 0.003) or relapse to organs (*P *= 0.006) other than the brain (Table 2). Furthermore, AI around the *PTEN *locus (CR1) was more common in HER2-negative brain metastases (seven of 11; 64%) compared with HER2-positive brain metastases (three of nine; 33%). The HER2 status was not inversely associated with AI at the *PTEN *locus in the primary tumors (19% and 25% in HER2-negative and HER2-positive primary tumors, respectively) (Figure 2).

The size of AI was often quite small (sometimes covering only one microsatellite marker) in many of the primary tumors, which could explain its rare detection by CGH. In general, however, allelic-imbalance detection was in good accordance with the results indicated by the CGH array (Figure 2).

Matched primary-tumor and brain-metastasis samples were available for AI analysis in four cases. Three cases showed identical aberration patterns at 10q, one normal and two AI, whereas in the fourth case, the AI was larger (that is, in the metastases, the distal marker was also affected by AI (Figure 2)).

In the primary tumors, AI at any locus was not significantly associated with any clinical or histopathologic factors (including hormone-receptor and HER2 status) other than brain relapse (Additional file 5).

### Differentially expressed genes at 10q loci

In *in silico *gene-expression analysis, we identified 49 transcripts residing in 10q22.3-qter that were significantly downregulated in the brain metastases samples compared with unmatched primary breast tumors without relapse. The first 6.3 MBp-large AI core region around markers D10S541 and D10S1765 (*PTEN*/CR1) contained nine different transcripts that were significantly downregulated. The tumor-suppressor gene *PTEN *was among these mostly uncharacterized genes. The second 6.4-MBp-large AI hot spot around the markers D10S173 and D10S190 (CR2) contained eight different transcripts, including the recently described tumor-suppressor gene *HTRA1 *[32] and three other genes (*GFRA1, HSPA12A, RGS10*) known to be involved in brain-related diseases (Additional file 6) [33-35].

To see whether a difference exists in PTEN expression patterns among different primary tumor patients with different relapse patterns, the GSE14020 data set was analyzed. SAM analysis revealed a significant downregulation of PTEN expression in patients with brain relapse compared with patients with bone relapse (Additional file 7). Interestingly, the PTEN expression was not significantly different between patients with lung or brain relapse (data not shown).

### PTEN mutation screening in brain metastases

The entire coding region in 10 brain-metastasis samples was sequenced for *PTEN *mutations. In addition, 10 samples were used to sequence exons 3 and 5 for mutations. Table 3 shows the three tumor samples (15%) with mutations. Two patients were carriers of a splice-site mutation. In one tumor, the cDNA lacked a complete exon 4. In the second tumor, both alleles were mutated, resulting in one product with a deletion of exons 4 to 6 and the second product with a complex translocation and duplication of exon 3. The deletion of exon 4 in the cDNA was shown to be caused by a 41-bp deletion in the intron 3-exon 4 junction of *PTEN*. Because of the complex nature of the translocations and deletions of both alleles in the second case, no clear sequencing product on genomic DNA could be obtained. The third mutation was detected in exon 5 (c.389G > T). This base-pair substitution causes an amino acid change of arginine to lysine (p.R130L) in the active-site pocket of the phosphatase domain, which is essential for catalysis [36]. This mutation was previously described as causing a loss of PTEN protein expression [37].

**Table 3:** *PTEN *mutations in brain metastases

Sample	Mutations in gDNA				Mutations in cDNA	AI result	CGH (PTEN)
				
	Exon3	Exon4	Exon5	Exon6			result
BrM-6	Wt	g.del[72586_72627]	wt	nd	c.[del1241_1284]	AI	Het. loss
							
BrM-7	Wt	No product	wt	wt	Allele 1: c.[del1241_1665] Allele 2: [del1524_1665; dup1196_1240, 950_1240con1196_1523]	AI	Het. loss
							
BrM-8	wt	nd	c.389C > A	nd	nd	Normal	nd

het. Loss, heterozygous loss; nd, not determined; wt, wild type.

## Discussion

Central nervous system metastases are a frequent complication of many solid tumors. Approximately 15% of all epithelial tumors metastasize to the brain, with incidence rates highly dependent on the primary tumor type. Whereas prostate cancer very rarely metastasizes to the brain (1% to 5%), small-cell lung cancer (40%) and breast cancer (15% to 20%) commonly metastasize to the brain [38]. Apparently, the brain microenvironment is especially permissive for the growth of disseminated tumor cells from some carcinomas but not from others. The mechanisms by which metastatic tumor cells adapt to the selection pressure exerted by the brain microenvironment are still unknown.

In this study, our aim was to identify putative molecular markers associated with the development of brain metastases in breast cancer. First, we screened for chromosomal aberrations by array CGH. The most prominent finding of the loss of 10q in brain metastases was validated in a larger study population, and the tumor-suppressor gene *PTEN *was found to be the potential target gene in this region.

Overall, the array CGH results of the primary breast tumors were in agreement with those described previously [39-41]. In general, the brain metastases showed aberration patterns similar to those of the primary tumors. However, in the brain metastases, a remarkably higher frequency of gains and losses was found at almost every chromosomal locus. Only a gain at 1p and a loss at 16q, described as being typical of luminal breast tumors and as markers of a favorable prognosis, were more common in the primary tumors [39-41]. This finding is not surprising, as most of the primary tumors were hormone receptor (HR) positive, whereas 38% of the brain metastases were HR negative. Statistically significant differences were found between the primary breast tumors and brain metastases at nine different loci on six different chromosomes.

The most significant differences were found at chromosomes 7 and 10. Chromosome 7 contains two regions, 7p22-p15 and 7p11.2, that were gained or amplified in more than 70% of the metastases and gained in only 3% to 13% of the primary tumors. Whereas the 22-Mbp region 7p22.1-p15.3 contains many genes, the second gained region on chromosome 7 contains only one gene, the epidermal growth factor receptor (*EGFR*); *EGFR *is a well-known and important gene in breast cancer initiation and progression [42]. Recently, Gaedcke *et al*. [43] reported a *de novo *protein expression of EGFR in the brain metastases of matched primary and metastatic breast cancer cases, and mouse models have shown the EGFR ligand HBEGF to be one of the key mediators of cancer-cell passage through the blood-brain barrier [5].

Most of the long arm of chromosome 10 was lost in 60% to 90% of the brain metastases, but only in none to 13% of the primary tumors. Numerous previously published large-array CGH datasets, also including locally advanced breast cancer, seldom showed a loss of 10q [39-41]. The loss of 10q is, in general, rarely seen in most epithelial tumors, whereas it is the most common aberration found in glioblastomas, and it also is common in other CNS malignancies [21,22]. Similarly, the gain of 7p is among the most frequently found gains in astrocytomas [44]. Both aberrations also are present in melanomas, which often metastasize to the brain (Additional file 8). The origin of these brain-specific aberrations in metastases could be explained by different hypotheses [45]. The first one is that only few cells carried these genetic alterations in the primary tumors, and thus they could not be detected when the tumors were analyzed in bulk. Subsequently, cells with these alterations selectively metastasized to the brain and formed the bulk of the metastases. Alternatively, these additional alterations might have occurred at the distant site of metastasis, and therefore represent *de novo *mutations that were not present in the primary tumors. The third scenario is that only a small fraction of the primary tumors contained these aberrations and that these tumors are specifically prone to relapse in the brain. Finally, another possibility is that several cells metastasize to the brain (for example, as a tumor-thrombus), but only those with alterations at 10q and/or 7p are able to survive in the brain environment, giving rise to metastases. Recently, by massively parallel DNA sequencing, Ding *et al*. [46] showed that metastases were indeed significantly enriched for shared mutations, which supports the last model.

Matched primary and metastatic tumor samples could be investigated in four cases by microsatellite analysis. Three cases showed identical aberration patterns, whereas in the fourth case, the AI imbalance was larger (that is, in the metastases, the distal marker was also affected by AI). Furthermore, primary tumor samples from patients in whom brain metastasis later developed showed a frequency of AI at 10q that was almost as high as in the brain metastases, but AI was rarely seen in primary tumors without brain relapse or other distant metastases. These results indicate that the loss of 10q does exist in a fraction of primary tumors with a high risk of developing brain metastases. The size of the aberration can expand in metastases and thus become more detectable by, for example, CGH. Because aberrations in 10q were not associated with any clinical factor other than brain relapse, this implies that loss of 10q is a specific marker of brain metastasis and is thus needed for the outgrowth of the breast tumor in the brain. However, this hypothesis must be validated in future studies, both functionally and on independent larger cohorts of patients.

The AIs were concentrated around two core regions, the first one around markers D10S1765 and D10S541 containing the *PTEN *locus, and the second around markers D10S1236 and D10S190 at 10q26. The *PTEN *gene located at 10q23.31 is a well-described tumor-suppressor gene, also in breast cancer; PTEN functions as an important tumor suppressor by negatively regulating the PI3K-mediated cell-signaling pathway [47]. The present microarray analysis showed that PTEN is significantly downregulated in brain metastases compared with nonmetastatic primary tumors. Furthermore, mutation screening of the *PTEN *gene in brain tumors showed that the frequency of the mutation was much higher (15%) in primary breast tumors (none to 5%) than previously described [48,49].

Several studies have shown that ERBB2/HER2 and the basal subtype of breast cancer are the predominant types of breast cancer that metastasize to the brain [43,50,51]. To avoid misleading results by analyzing unmatched samples, the primary tumor sample cohort was matched for the main clinicopathologic characteristics in our AI analysis. When we classified the primary tumors as being ER/PR-positive, triple-negative, and HER2-positive tumors, no association was found between PTEN status (or loss of 10q) and breast cancer subtype, indicating that loss of PTEN is an independent predictor of brain metastases. In a recent publication, the protein expression of PTEN was analyzed in 54 brain metastasis samples from breast cancer patients [52]; no correlation was found between PTEN loss and subtype in this study. Furthermore, a high concordance rate (83%) of PTEN expression was found among the 12 matched pairs. The authors also investigated the role of PTEN expression in primary breast cancer progression by using *in silico *expression datasets with 855 patients [52]. They showed that, in all patients, lower levels of PTEN expression were associated with a poor prognosis and shorter time to brain recurrence, irrespective of hormone-receptor and HER2 status after 5 years. Also, this analysis was independent of subtype. In addition, we found, by using the same data set, a significant downregulation of PTEN expression among primary tumor patients with brain relapse compared with patients with bone relapse, but not to the lung. This finding is in line with the findings from Bos *et al*. [4,5], who found a significant overlap of brain with lung-relapse signature, but not with the bone signature, which argues for the important role of environment interaction in metastasis formation in different organs.

Interestingly, the two predominant genes derived from this breast cancer study on brain metastases also play a prominent role in the development and progression of primary brain tumors. The *PTEN *gene is one of the key tumor-suppressor genes found in primary glioblastomas, and it is often (15% to 40%) silenced through mutations (reviewed in [21]). Interestingly, also in glioblastomas, PTEN inactivation does not seem to be required for tumor initiation, but its loss is a hallmark for progression to highly malignant cancer [53]. Together with *EGFR *amplification, the loss of PTEN is the most frequent alteration observed in primary glioblastomas. Thus, these two genes, which are both involved in the PI3K kinase pathway, may play a key role in the growth of malignant cells in the brain environment and therefore might be suitable targets for therapeutic intervention.

## Conclusions

In conclusion, the present results show that brain metastases in breast cancer often carry very complex genomic aberration patterns. Nevertheless, certain target genes, such as *PTEN *and *EGFR*, are predominantly affected and might therefore play an important role in brain metastasis formation. The genetic analyses of these genes might contribute to defining a subgroup of breast cancer patients who are at high risk of developing brain metastases. Moreover, increasing knowledge about the genetics of brain metastasis with regard to therapeutic targets and pathways may eventually lead to new antimetastatic strategies.

## Abbreviations

AI: allelic imbalance; CGH: comparative genetic hybridization; CNS: central nervous system; CR: core region; fdr: false discovery rate; FISH: fluorescence *in situ *hybridization; HR: hormone receptor.

## Competing interests

The authors declare that they have no competing interests. The work was funded by DFG (PA 341/15-2, HW, KP) and EU (DISMAL; HW, KE, KP, MZ), ERC Advanced Investigator grant no. 269081 (DISSECT, KP), VFK Krebsforschung GmbH (TIME, HW, KE, KP).

## Authors' contributions

HW, ND, LU, MW, IH, and KY carried out the molecular genetic studies, CB, DK, and MZ carried out the statistical analyses. DK and BY performed the microarray analyses. HW, KL, NW, GS, MG, VM, and KP participated in the design of the study and coordination and helped to draft the manuscript. All authors read and approved the final manuscript.

## Supplementary Material

Additional file 1 **Table S1: Clinicopathologic characteristics of the patients**. Click here for file

Additional file 2 **Table S2: Primers used for the AI analysis and *PTEN *mutation analysis**. Click here for file

Additional file 3 **Table S3: High-level amplifications detected in primary and metastatic breast tumors**. Click here for file

Additional file 4 **Figure S1: Chromosome 10q AI hot-spot region detection**. Click here for file

Additional file 5 **Table S4: 10q allelic imbalances and association to clinical factors in primary tumors**. Click here for file

Additional file 6 **Table S5: Differentially expressed genes at 10q between primary tumors and brain metastases**. Genes found significantly differentially expressed between primary tumors and brain metastases by using SAM analysis. Genes marked in bold reside in regions defined as hot spots by using the microsatellite analysis. Click here for file

Additional file 7 **Table S6: Genes significantly differently expressed between primary breast cancer patients with bone and brain relapse**. Click here for file

Additional file 8 **Figure S2**. CGH copy-number patterns in different tumor entities showing a high additional gain of 7p and loss of 10q. Frequencies and plots of gains and losses were retrieved from a CGH data base progenetix http://www.progenetix.net. Click here for file

## References

[B1] Schouten LJ Rutten J Huveneers HA Twijnstra A Incidence of brain metastases in a cohort of patients with carcinoma of the breast, colon, kidney, and lung and melanoma Cancer 2002 94 2698 2705 10.1002/cncr.10541 12173339

[B2] Leyland-Jones B Human epidermal growth factor receptor 2-positive breast cancer and central nervous system metastases J Clin Oncol 2009 27 5278 5286 10.1200/JCO.2008.19.8481 19770385

[B3] Gril B Evans L Palmieri D Steeg PS Translational research in brain metastasis is identifying molecular pathways that may lead to the development of new therapeutic strategies Eur J Cancer 2009 46 1204 1210 20303257 10.1016/j.ejca.2010.02.033 PMC2858326

[B4] Pantel K Brakenhoff RH Brandt B Detection, clinical relevance and specific biological properties of disseminating tumour cells Nat Rev Cancer 2008 5 329 340 18404148 10.1038/nrc2375

[B5] Bos PD Zhang XH Nadal C Shu W Gomis RR Nguyen DX Minn AJ van de Vijver MJ Gerald WL Foekens JA Massague J Genes that mediate breast cancer metastasis to the brain Nature 2009 459 1005 1009 10.1038/nature08021 19421193 PMC2698953

[B6] Ramaswamy S Ross KN Lander ES Golub TR A molecular signature of metastasis in primary solid tumors Nat Genet 2003 33 49 54 10.1038/ng1060 12469122

[B7] Weigelt B Glas AM Wessels LF Witteveen AT Peterse JL van't Veer LJ Gene expression profiles of primary breast tumors maintained in distant metastases Proc Natl Acad Sci USA 2003 100 15901 15905 10.1073/pnas.2634067100 14665696 PMC307665

[B8] Dupont VN Gentien D Oberkampf M De Rycke Y Blin N A gene expression signature associated with metastatic cells in effusions of breast carcinoma patients Int J Cancer 2007 121 1036 1046 10.1002/ijc.22775 17450528

[B9] Palmieri D Fitzgerald D Shreeve SM Hua E Bronder JL Weil RJ Davis S Stark AM Merino MJ Kurek R Mehdorn HM Davis G Steinberg SM Meltzer PS Aldape K Steeg PS Analyses of resected human brain metastases of breast cancer reveal the association between up-regulation of hexokinase 2 and poor prognosis Mol Cancer Res 2009 7 1438 1445 10.1158/1541-7786.MCR-09-0234 19723875 PMC2746883

[B10] Suzuki M Tarin D Gene expression profiling of human lymph node metastases and matched primary breast carcinomas: clinical implications Mol Oncol 2007 1 172 180 10.1016/j.molonc.2007.03.005 19383293 PMC5543892

[B11] Hao X Sun B Hu L Lahdesmaki H Dunmire V Feng Y Zhang SW Wang H Wu C Fuller GN Symmans WF Shmulevich I Zhang W Differential gene and protein expression in primary breast malignancies and their lymph node metastases as revealed by combined cDNA microarray and tissue microarray analysis Cancer 2004 100 1110 1122 10.1002/cncr.20095 15022276

[B12] Wu JM Fackler MJ Halushka MK Molavi DW Taylor ME Teo WW Griffin C Fetting J Davidson NE De Marzo AM Hicks JL Chitale D Ladanyi M Sukumar S Argani P Heterogeneity of breast cancer metastases: comparison of therapeutic target expression and promoter methylation between primary tumors and their multifocal metastases Clin Cancer Res 2008 14 1938 1946 10.1158/1078-0432.CCR-07-4082 18381931 PMC2965068

[B13] Santos SC Cavalli IJ Ribeiro EM Urban CA Lima RS Bleggi-Torres LF Rone JD Haddad BR Cavalli LR Patterns of DNA copy number changes in sentinel lymph node breast cancer metastases Cytogenet Genome Res 2008 122 16 21 10.1159/000151311 18931481

[B14] Wang C Iakovlev VV Wong V Leung S Warren K Iakovleva G Arneson NC Pintilie M Miller N Youngson B McCready DR Done SJ Genomic alterations in primary breast cancers compared with their sentinel and more distal lymph node metastases: an aCGH study Genes Chromosomes Cancer 2009 48 1091 1101 10.1002/gcc.20711 19760610

[B15] Morandi L Marucci G Foschini MP Cattani MG Pession A Riva C Eusebi V Genetic similarities and differences between lobular in situ neoplasia (LN) and invasive lobular carcinoma of the breast Virchows Arch 2006 449 14 23 10.1007/s00428-006-0192-7 16612623

[B16] Torres L Ribeiro FR Pandis N Andersen JA Heim S Teixeira MR Intratumor genomic heterogeneity in breast cancer with clonal divergence between primary carcinomas and lymph node metastases Breast Cancer Res Treat 2007 102 143 155 10.1007/s10549-006-9317-6 16906480

[B17] Nishizaki T DeVries S Chew K Goodson WH Ljung BM Thor A Waldman FM Genetic alterations in primary breast cancers and their metastases: direct comparison using modified comparative genomic hybridization Genes Chromosomes Cancer 1997 19 267 272 10.1002/(SICI)1098-2264(199708)19:4<267::AID-GCC9>3.0.CO;2-V 9258662

[B18] Petersen I Hidalgo A Petersen S Schluns K Schewe C Pacyna-Gengelbach M Goeze A Krebber B Knosel T Kaufmann O Szymas J von Deimling A Chromosomal imbalances in brain metastases of solid tumors Brain Pathol 2000 10 395 401 10885658 10.1111/j.1750-3639.2000.tb00271.x PMC8098540

[B19] Hampl M Hampl JA Schwarz P Frank S Hahn M Schackert G Saeger HD Schackert HK Accumulation of genetic alterations in brain metastases of sporadic breast carcinomas is associated with reduced survival after metastasis Invasion Metastasis 1998 18 81 95 10.1159/000024501 10364688

[B20] Stoecklein NH Klein CA Genetic disparity between primary tumours, disseminated tumour cells, and manifest metastasis Int J Cancer 2010 126 589 598 10.1002/ijc.24916 19795462

[B21] Ohgaki H Kleihues P Genetic alterations and signaling pathways in the evolution of gliomas Cancer Sci 2009 100 2235 2241 10.1111/j.1349-7006.2009.01308.x 19737147 PMC11159448

[B22] Lamszus K Meningioma pathology, genetics, and biology J Neuropathol Exp Neurol 2004 63 275 286 15099018 10.1093/jnen/63.4.275

[B23] De Witt Hamer PC Van Tilborg AA Eijk PP Sminia P Troost D Van Noorden CJ Ylstra B Leenstra S The genomic profile of human malignant glioma is altered early in primary cell culture and preserved in spheroids Oncogene 2008 27 2091 2096 10.1038/sj.onc.1210850 17934519

[B24] Wrage M Ruosaari S Eijk PP Kaifi JT Hollmen J Yekebas EF Izbicki JR Brakenhoff RH Streichert T Riethdorf S Glatzel M Ylstra B Pantel K Wikman H Genomic profiles associated with early micrometastasis in lung cancer: relevance of 4q deletion Clin Cancer Res 2009 15 1566 1574 10.1158/1078-0432.CCR-08-2188 19208797

[B25] van den Ijssel P Tijssen M Chin SF Eijk P Carvalho B Hopmans E Holstege H Bangarusamy DK Jonkers J Meijer GA Caldas C Ylstra B Human and mouse oligonucleotide-based array CGH Nucleic Acids Res 2005 33 e192 10.1093/nar/gni191 16361265 PMC1316119

[B26] Hupe P Stransky N Thiery JP Radvanyi F Barillot E Analysis of array CGH data: from signal ratio to gain and loss of DNA regions Bioinformatics 2004 20 3413 3422 10.1093/bioinformatics/bth418 15381628

[B27] ArrayExpress http://www.ebi.ac.uk/arrayexpress/experiments/E-MTAB-354

[B28] Danielsen SA Lind GE Bjornslett M Meling GI Rognum TO Heim S Lothe RA Novel mutations of the suppressor gene PTEN in colorectal carcinomas stratified by microsatellite instability and TP53 mutation status Hum Mutat 2008 29 E252 262 10.1002/humu.20860 18781614

[B29] CGHtest http://www.few.vu.nl/~mavdwiel/CGHtest.html

[B30] van de Wiel MA Smeets SJ Brakenhoff RH Ylstra B CGHMultiArray: exact P-values for multi-array comparative genomic hybridization data Bioinformatics 2005 21 3193 3194 10.1093/bioinformatics/bti489 15879450

[B31] Benjamini Y Hochberg Y Controlling the false discovery rate: a practical and powerful approach to multiple testing J R Stat Soc 1995 57 289 300

[B32] Zurawa-Janicka D Skorko-Glonek J Lipinska B HtrA proteins as targets in therapy of cancer and other diseases Expert Opin Ther Targets 2010 14 665 679 10.1517/14728222.2010.487867 20469960

[B33] Wan G Too HP A specific isoform of glial cell line-derived neurotrophic factor family receptor alpha 1 regulates RhoA expression and glioma cell migration J Neurochem 2010 115 759 770 10.1111/j.1471-4159.2010.06975.x 20807316

[B34] Pongrac JL Middleton FA Peng L Lewis DA Levitt P Mirnics K Heat shock protein 12A shows reduced expression in the prefrontal cortex of subjects with schizophrenia Biol Psychiatry 2004 56 943 950 10.1016/j.biopsych.2004.09.005 15601604

[B35] Lee JK McCoy MK Harms AS Ruhn KA Gold SJ Tansey MG Regulator of G-protein signaling 10 promotes dopaminergic neuron survival via regulation of the microglial inflammatory response J Neurosci 2008 28 8517 8528 10.1523/JNEUROSCI.1806-08.2008 18716210 PMC2739568

[B36] Lee JO Yang H Georgescu MM Di Cristofano A Maehama T Shi Y Dixon JE Pandolfi P Pavletich NP Crystal structure of the PTEN tumor suppressor: implications for its phosphoinositide phosphatase activity and membrane association Cell 1999 99 323 334 10.1016/S0092-8674(00)81663-3 10555148

[B37] Byron SA Gartside MG Wellens CL Mallon MA Keenan JB Powell MA Goodfellow PJ Pollock PM Inhibition of activated fibroblast growth factor receptor 2 in endometrial cancer cells induces cell death despite PTEN abrogation Cancer Res 2008 68 6902 6907 10.1158/0008-5472.CAN-08-0770 18757403

[B38] Nelson J von Deimling A Kleihues P, Cavenee WK Metastatic tumors Pathology and Genetics of Tumors of the Nervous System 1997 16 Lyon: International Agency Research on Cancer 200 202

[B39] Bergamaschi A Kim YH Wang P Sorlie T Hernandez-Boussard T Lonning PE Tibshirani R Borresen-Dale AL Pollack JR Distinct patterns of DNA copy number alteration are associated with different clinicopathological features and gene-expression subtypes of breast cancer Genes Chromosomes Cancer 2006 45 1033 1040 10.1002/gcc.20366 16897746

[B40] Chin K DeVries S Fridlyand J Spellman PT Roydasgupta R Kuo WL Lapuk A Neve RM Qian Z Ryder T Chen F Feiler H Tokuyasu T Kingsley C Dairkee S Meng Z Chew K Pinkel D Jain A Ljung BM Esserman L Albertson DG Waldman FM Gray JW Genomic and transcriptional aberrations linked to breast cancer pathophysiologies Cancer Cell 2006 10 529 541 10.1016/j.ccr.2006.10.009 17157792

[B41] Nordgard SH Johansen FE Alnaes GI Bucher E Syvanen AC Naume B Borresen-Dale AL Kristensen VN Genome-wide analysis identifies 16q deletion associated with survival, molecular subtypes, mRNA expression, and germline haplotypes in breast cancer patients Genes Chromosomes Cancer 2008 47 680 696 10.1002/gcc.20569 18398821

[B42] Foley J Nickerson NK Nam S Allen KT Gilmore JL Nephew KP Riese DJ EGFR signaling in breast cancer: bad to the bone Semin Cell Dev Biol 2010 21 951 960 10.1016/j.semcdb.2010.08.009 20813200 PMC2991402

[B43] Gaedcke J Traub F Milde S Wilkens L Stan A Ostertag H Christgen M von Wasielewski R Kreipe HH Predominance of the basal type and HER-2/neu type in brain metastasis from breast cancer Mod Pathol 2007 20 864 870 10.1038/modpathol.3800830 17541441

[B44] Schlegel J Scherthan H Arens N Stumm G Kiessling M Detection of complex genetic alterations in human glioblastoma multiforme using comparative genomic hybridization J Neuropathol Exp Neurol 1996 55 81 87 10.1097/00005072-199601000-00008 8558174

[B45] Pantel K Brakenhoff RH Dissecting the metastatic cascade Nat Rev Cancer 2004 4 448 456 10.1038/nrc1370 15170447

[B46] Ding L Ellis MJ Li S Larson DE Chen K Wallis JW Harris CC McLellan MD Fulton RS Fulton LL Abbott RM Hoog J Dooling DJ Koboldt DC Schmidt H Kalicki J Zhang Q Chen L Lin L Wendl MC McMichael JF Magrini VJ Cook L McGrath SD Vickery TL Appelbaum E Deschryver K Davies S Guintoli T Crowder R Tao Y Snider JE Smith SM Dukes AF Sanderson GE Pohl CS Delehaunty KD Fronick CC Pape KA Reed JS Robinson JS Hodges JS Schierding W Dees ND Shen D Locke DP Wiechert ME Eldred JM Peck JB Oberkfell BJ Lolofie JT Du F Hawkins AE O'Laughlin MD Bernard KE Cunningham M Elliott G Mason MD Thompson DM Jr Ivanovich JL Goodfellow PJ Perou CM Weinstock GM Aft R Watson M Ley TJ Wilson RK Mardis ER Genome remodelling in a basal-like breast cancer metastasis and xenograft Nature 2010 464 999 1005 10.1038/nature08989 20393555 PMC2872544

[B47] Hollander MC Blumenthal GM Dennis PA PTEN loss in the continuum of common cancers, rare syndromes and mouse models Nat Rev Cancer 2011 11 289 301 10.1038/nrc3037 21430697 PMC6946181

[B48] Saal LH Holm K Maurer M Memeo L Su T Wang X Yu JS Malmstrom PO Mansukhani M Enoksson J Hibshoosh H Borg A Parsons R PIK3CA mutations correlate with hormone receptors, node metastasis, and ERBB2, and are mutually exclusive with PTEN loss in human breast carcinoma Cancer Res 2005 65 2554 2559 10.1158/0008-5472-CAN-04-3913 15805248

[B49] Stemke-Hale K Gonzalez-Angulo AM Lluch A Neve RM Kuo WL Davies M Carey M Hu Z Guan Y Sahin A Symmans WF Pusztai L Nolden LK Horlings H Berns K Hung MC van de Vijver MJ Valero V Gray JW Bernards R Mills GB Hennessy BT An integrative genomic and proteomic analysis of PIK3CA, PTEN, and AKT mutations in breast cancer Cancer Res 2008 68 6084 6091 10.1158/0008-5472.CAN-07-6854 18676830 PMC2680495

[B50] Smid M Wang Y Zhang Y Sieuwerts AM Yu J Klijn JG Foekens JA Martens JW Subtypes of breast cancer show preferential site of relapse Cancer Res 2008 68 3108 3114 10.1158/0008-5472.CAN-07-5644 18451135

[B51] Shao MM Liu J Vong JS Niu Y Germin B Tang P Chan AW Lui PC Law BK Tan PH Tse GM A subset of breast cancer predisposes to brain metastasis Med Mol Morphol 2011 44 15 20 10.1007/s00795-010-0495-2 21424932

[B52] Adamo B Deal AM Burrows E Geradts J Hamilton E Blackwell KL Livasy C Fritchie K Prat A Harrell JC Ewend MG Carey LA Miller CR Anders CK Phosphatidylinositol 3-kinase (PI3K) pathway activation in breast cancer brain metastases Breast Cancer Res 2011 13 R125 10.1186/bcr3071 22132754 PMC3326567

[B53] Koul D PTEN signaling pathways in glioblastoma Cancer Biol Ther 2008 7 1321 1325 10.4161/cbt.7.9.6954 18836294

