# Supplementary material for: Relevance of PTEN loss in brain metastasis formation in breast cancer patients
Source: Breast Cancer Res. 2012 Mar 19;14(2):R49. doi: 10.1186/bcr3150 (PMC3446383; doi:10.1186/bcr3150)
Supplement: Additional file 8 — Figure S2. CGH copy-number patterns in different tumor entities showing a high additional gain of 7p and loss of 10q. Frequencies and plots of gains and losses were retrieved from a CGH data base progenetix http://www.progenetix.net. [file bcr3150-S8.PPT]

## Slide 1
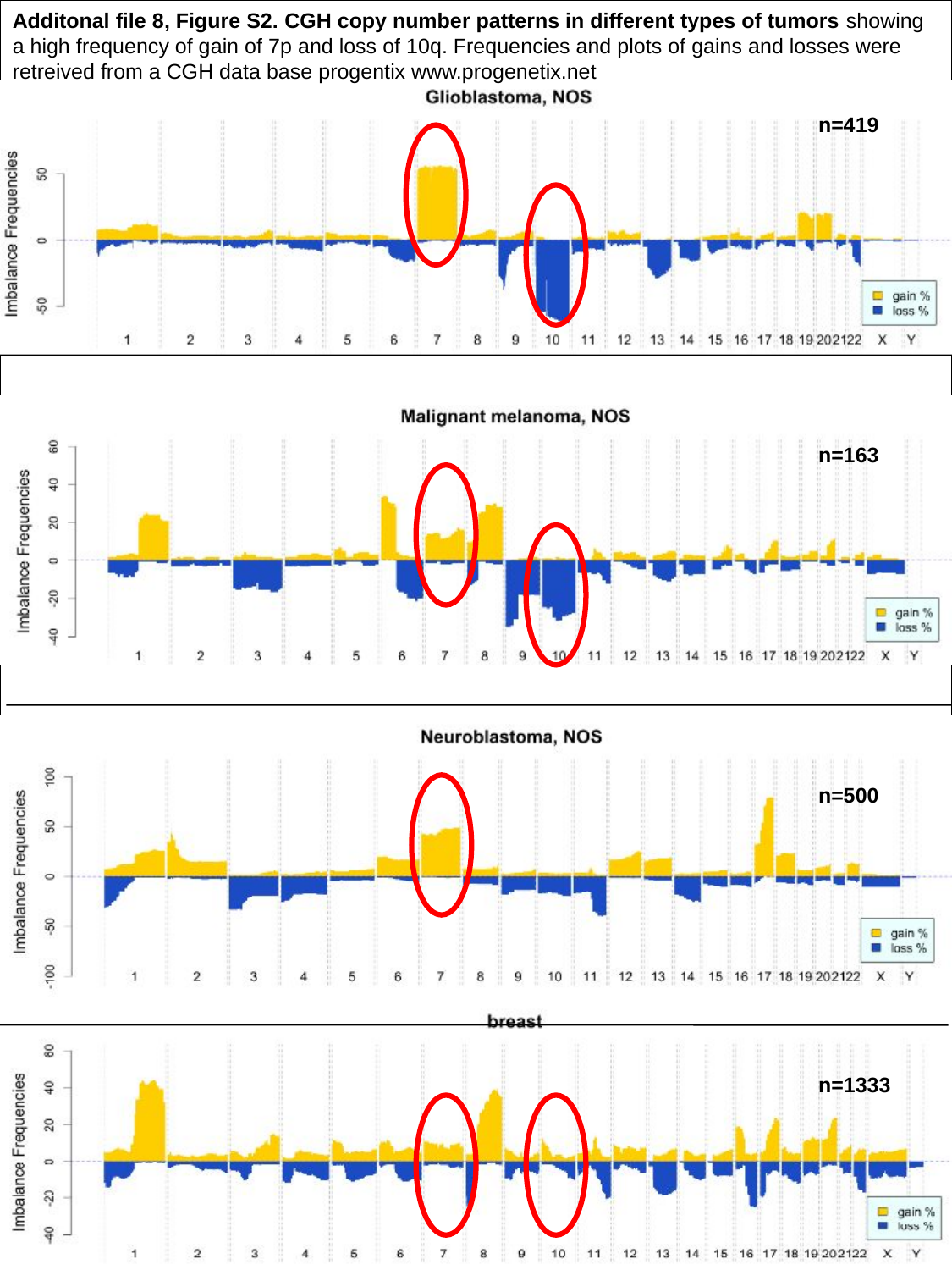

Additonal file 8, Figure S2. CGH copy number patterns in different types of tumors showing a high frequency of gain of 7p and loss of 10q. Frequencies and plots of gains and losses were retreived from a CGH data base progentix www.progenetix.net
n=419
n=163
n=500
n=1333
